# Successful management of synchronous recurrent breast carcinoma with chronic myelogenous leukemia: a case report

**DOI:** 10.1186/s13256-016-1180-4

**Published:** 2017-01-10

**Authors:** Choukri Elm’hadi, Mohamed Reda Khmamouche, Rachid Tanz, Mehdi Toreis, ElMehdi Mahtat, Mohammed Allaoui, Mohammed Oukabli, Nezha Messaoudi, Hassan Errihani, Mohammed Ichou

**Affiliations:** 1 Medical Oncology Department, Mohammed V Military Teaching Hospital of Rabat, Rabat, Morocco; 2 Clinical Hematology Department, Mohammed V Military Teaching Hospital of Rabat, Rabat, Morocco; 3 Department of Pathology, Mohammed V Military Teaching Hospital of Rabat, Rabat, Morocco; 4 Laboratory of Hematology, Mohammed V Military Teaching Hospital of Rabat, Rabat, Morocco; 5 Medical Oncology Department, National Institute of Oncology Sidi Mohamed Ben Abdellah, Rabat, Morocco; 6 School of Medicine and Pharmacy, University Mohamed V, Souissi, Rabat, Morocco

**Keywords:** Relapse, Breast cancer, Chronic myeloid leukemia, Management

## Abstract

**Background:**

Survival is increasing after early breast cancer revealing frequent relapses and possibility of developing secondary malignancies. The concomitant occurrence of these two events is exceptionally disastrous and lethal. We report a case of a Moroccan woman who was successfully managed for synchronous recurrent breast carcinoma and chronic myelogenous leukemia.

**Case presentation:**

A 42-year-old Moroccan woman was diagnosed with localized breast carcinoma in 2008. She received six cycles of an adjuvant chemotherapy regimen, radiation therapy and hormonal therapy by tamoxifen. After completion of 5 years of tamoxifen our patient reported asthenia; a physical examination found hepatomegaly, massive splenomegaly measuring 21 cm and supraclavicular lymphadenopathy. The staging showed lung and liver metastases. Morphology and immunohistochemical profile of this metastasis identified an adenocarcinoma of mammary origin. In parallel, the diagnosis of chronic myeloid leukemia was suspected because of the presence of a leukocytosis at 355 × 10^9^/L, with circulating blasts of 4%. Chronic myeloid leukemia was confirmed by a bone marrow biopsy with the presence of Ph chromosome on cytogenetical analysis.

Daily imatinib was ordered concurrently with chemotherapy-type docetaxel. The metastases were stable after nine courses of chemotherapy. Due to breast cancer progression 4 months later, bevacizumab and capecitabine were introduced.

A major molecular response was achieved after 12 and 18 months. She has now completed 2 years of follow-up, still on a major molecular response, and is undergoing imatinib and capecitabine treatment.

**Conclusions:**

Leukocytosis in breast cancer patients can reveal chronic myeloid leukemia. It may warrant a workup to find the underlying etiology, which could include a secondary hematological malignancy.

## Background

Breast cancer is the most frequently diagnosed cancer among women [1]. Due to early detection of breast cancer and effective therapeutic regimens, survival is increasing but it is associated with frequent relapses and the possibility of developing secondary malignancies [2]. The concomitant occurrence of these two events is exceptionally disastrous and lethal in this population. Though a rare occurrence, it is possible to see secondary leukemias in breast cancer survivors. Data on the risks of chronic myelogenous in breast cancers survivors after adjuvant therapy are sparse. We report a case of a Moroccan woman who presented with recurrent breast cancer concurrently diagnosed with chronic myelogenous leukemia (CML).

## Case presentation

A 42-year-old Moroccan woman was diagnosed with breast cancer in 2008 and underwent right modified radical mastectomy. The tumor was infiltrating ductal carcinoma pT2N1M0 with 2 out of 12 lymph nodes positive. The tumor expressed hormone receptors (estrogen receptor was 90% and progesterone receptor was 70%) and the HercepTest result was negative. Her complete blood count showed a hemoglobin level of 13.7 g/dL (normal range: 12–16 g/dL), a platelet count of 250 × 10^9^/L (normal range: 150–400 × 10^9^/L), a leukocytes count of 7.3 × 10^9^/L (normal range: 4–10 × 10^9^/L) and a neutrophils count of 5.1 × 10^9^/L (normal range: 1.5–7 × 10^9^/L). She received six cycles of adjuvant 5-fluorouracil (500 mg/m^2^), epirubicin (100 mg/m^2^) and cyclophosphamide (500 mg/m^2^) (FEC100). The total dose was 960 mg of epirubicin and 4800 mg of cyclophosphamide. Adjuvant chemotherapy was followed by radiation therapy to her chest wall and ipsilateral axillary lymph node metastasis. She was placed on tamoxifen for 5 years.

**Fig. 1:**
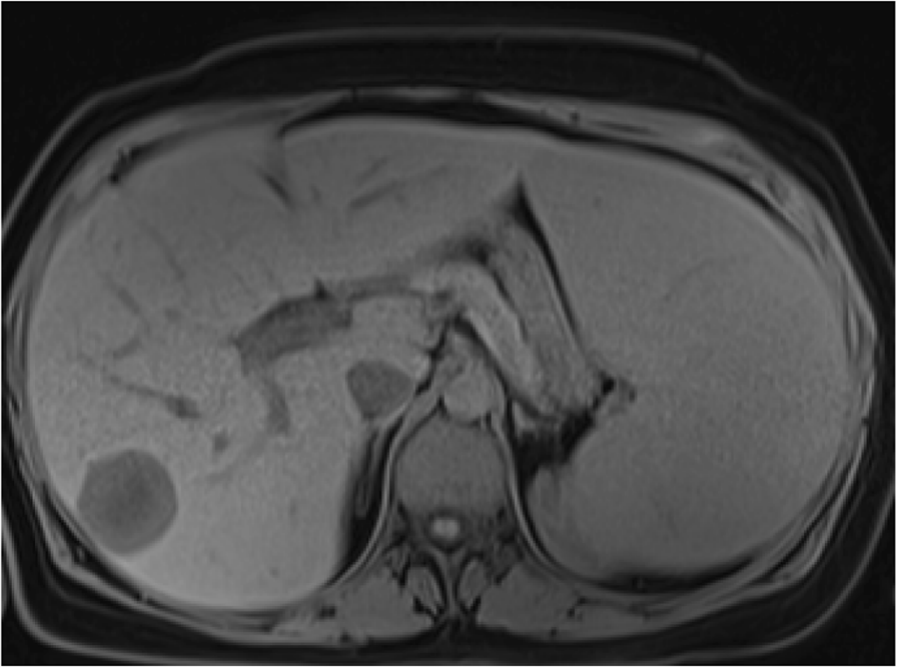
An abdominal magnetic resonance imaging scan showing a nodule in liver segment VII, hypointense on T1, measuring 37 mm × 32 mm

**Fig. 2:**
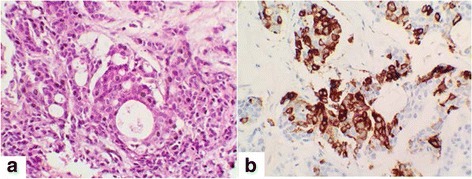
Moderately differentiated adenocarcinomatous proliferation: **a**: hematoxylin and eosin staining ×400 **b** Intense expression of mammaglobin by tumor cells

After completion of 5 years of tamoxifen our patient reported asthenia; a physical examination found hepatomegaly, splenomegaly extending into the umbilicus measuring 21 cm and supraclavicular lymphadenopathy measuring 2 cm, painless and mobile. Her cancer antigen 15-3 (CA15-3) level was 80 UI/mL (normal value less than 25 UI/mL). A thoracoabdominal computed tomography scan showed lung metastases with a hypodense nodule in segment VII of the liver characterized as a metastasis on a magnetic resonance imaging (MRI) scan (Fig. 1). A biopsy of this nodule was performed. Morphology and an immunohistochemical profile of this metastasis reveal an adenocarcinoma of mammary origin expressing cytokeratin 7 and mammaglobin (Fig. 2). The tumor was triple negative (TN).

**Fig. 3:**
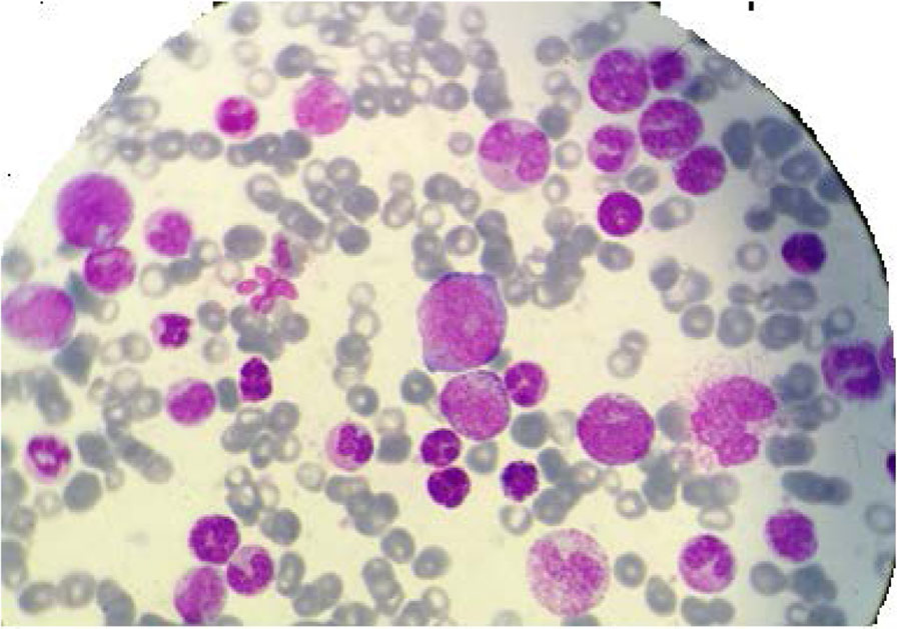
Myelogram showing a myeloproliferative disorder: rare megakaryocytes with very rich granulocyte elements

Concurrently, our patient’s blood count showed a hyperleukocytosis at 355 × 10^9^/L with a neutrophil count of 152 × 10^9^/L, her hemoglobin level was 10.6 g/dL and platelet count 264 × 10^9^/L. A peripheral smear showed myeloblasts at 4%, promyelocytic at 11%, neutrophilic metamyelocytes at 15%, and neutrophilic myelocytes at 19%. Further investigation by bone marrow aspiration evoked a myeloproliferative disorder (Fig. 3); the marrow was very rich with granulocyte elements as blasts (13%), eosinophils (4%) and basophils (4%). Megakaryocytes were rare and multilineage dysplasia signs were observed. Medullary karyotype showed the presence of the translocation (9; 22) (q34; q11) over 20 mitoses analyzed and BCR-ABL by real-time reverse transcriptase polymerase chain reaction (RT-PCR) quantitative assay was positive. She was finally diagnosed as synchronous CML and metastatic relapse of breast cancer.

Our patient then received a dose of 400 mg of imatinib daily taken as four 100 mg tablets, concurrently with chemotherapy-type docetaxel of 100 mg/m^2^ every 21 days without limiting hematologic toxicity. Liver and lung metastases were stable after nine courses of chemotherapy. The breast cancer progressed 4 months later due to the emergence of new lung metastases, then a treatment based on bevacizumab and capecitabine was introduced.

A major molecular response was achieved after 12 months using the RT-PCR method and maintained after 18 months. She has now completed 2 years of follow-up, still on a major molecular response, and is undergoing imatinib and capecitabine therapy.

## Discussion

Breast tumors are very heterogeneous and can be classified in three main groups based on their molecular profile: luminal cancers that express estrogen and/or progesterone receptors; human epidermal growth factor receptor 2 (HER2)-positive cancers that express the tyrosine kinase receptor ERBB2; and triple-negative (TN) cancers in which none of these receptors is detected. TN breast cancers are the most aggressive and have the poor prognosis due to the lack of specific therapies [3].

Treatment of breast cancer typically involves breast-conserving surgery followed by locoregional radiotherapy with or without systemic therapy adjuvant combination chemotherapy regimens containing alkylating agents, such as cyclophosphamide, methotrexate, and 5-fluorouracil (CMF) and anthracyclines such as doxorubicin and epirubicin. This treatment has been revolutionized by the advent of taxanes, targeted therapies, and endocrine therapy.

Leukemia is an unusual event in the natural history of breast cancer, though its association with breast cancer therapy has been reported. Several types of leukemia can be observed such as acute lymphocytic leukemia, acute myeloid leukemia (AML), CML, chronic myelomonocytic leukemia, myelodysplastic syndrome (MDS) and T cell prolymphocytic leukemia. Data on second hematological malignancies after treatment for breast cancer mostly describe myeloid malignancies/MDS in contrast to lymphoid malignancies [4].

CML is a slow-growing tumor of white blood cells, characterized by an unregulated growth of the myeloid precursor cells and its accumulation in the bone marrow and the lymphoid organs. CML is more frequent in men between 25 and 60 years. These tumors occur as a carcinogenic effect of ionizing radiation and show frequency among atomic bomb survivors [5]. Recently, the translocation of regions of the BCR and ABL genes to form a BCR-ABL fusion gene has been clearly documented in 90% of CML patients. This phenomenon is a reciprocal translocation termed t (9; 22), which forms the Philadelphia (Ph) chromosome. BCR-ABL is an oncogene that overexpresses a tyrosine-kinase protein that stimulates the leukemic growth of myoblasts; it has been successfully targeted by a tyrosine kinase inhibitor such as imatinib [6].

The results of randomized clinical trials have suggested that patients with primary breast carcinoma have an increased risk of developing leukemia. But this risk is not well characterized [7]. Increase in risk is attributable to adjuvant therapy, especially anthracycline and alkylating agent dose intensification, and perhaps to concomitant radiotherapy use [8]. The equivocal risk of leukemia associated with current chemotherapy regimens should not justify the therapeutic de-escalation to prevent breast carcinoma recurrence. Also, advances in radiation technology with less bone marrow exposure significantly decreased risk of CML [9].

CML was mostly described after adjuvant treatment of breast cancer, as in patients treated for lymphoma, testicular cancer, and colorectal cancer [10].Moreover, only two studies showed an increasing specific risk of CML after breast cancer treatment [9, 10]. The interval between the adjuvant treatment of breast cancer and CML was 4.7 years and this risk persisted over 25 years after breast cancer diagnosis [9]. Patients tended to be younger than those with CML diagnosed in the general population [9]. Cumulative doses were 240 mg/m2 for anthracycline and 4800 mg/m2 for alkylator [9]. In our case, cumulative doses of epirubicin (960 mg/m2) and cyclophosphamide (4800 mg/m2) may explain the occurrence of CML.

Only one case of simultaneous occurrence of CML and breast carcinoma has been reported in the literature but CML revealing relapse of breast carcinoma has not been reported [11].

The occurrence of CML after breast cancer treated with surgery alone suggests inherent genetic predisposition of the patient to the genesis of second malignancies including leukemias [7].

The exact pathogenesis of synchronous occurrence of CML and TN breast carcinoma requires further investigation. Mutations in BRCA1 and BRCA2 increase the risk of female breast and ovarian cancers. They tend to develop at younger ages and are more likely to develop TN breast cancers. There are similarities between BRCA1, BRCA2 and BCR-ABL [12]. BRCA1 expression is downregulated in CML cells. It becomes nearly undetectable during the chronic phase and blast crisis. Recently, ABL kinase has also been implicated in TN breast cancer development and progression [13]. In our patient, leukemogenic factors implicated are higher cumulative doses of alkylating agent, anthracycline, and radiotherapy. Tamoxifen has not been associated with leukemia risk [14]. The interval between the adjuvant treatment of breast cancer and CML was 5 years and the patient was younger. Genetic testing of BRCA is important to this case but was not performed. The therapeutic efficiency obtained in our patient may be explained by the sensitivity of cell lines to the action of ABL inhibitors [13], and the use of bevacizumab, a monoclonal antibody to vascular endothelial growth factor (VEGF) which potentiates the action of imatinib, was like that described in many solid cancers, and inhibits VEGF involved in the initiation and development of CML [15].

Leukoerytroblastic reaction can occur in breast cancer, mainly in marrow metastasis [16]. Marrow involvement should be suspected in patients with bone metastases and otherwise unexplained cytopenia and confirmed by a positive bone marrow aspirate/trephine biopsy. In the absence of these elements, like in our patient, this diagnosis should be excluded.

## Conclusions

Leukocytosis in breast cancer patients can reveal chronic myeloid leukemia if a medullary metastatic localization is excluded. It may warrant a workup to find the underlying etiology, which could include a secondary hematological malignancy. Optimization of treatment mainly in young patients can prolong survival.

## Abbreviations

AML: acute myeloid leukemia
CA15-3: cancer antigen 15-3
CMF: cyclophosphamide, methotrexate, and 5-fluorouracil
CML: chronic myelogenous leukemia
FEC: fluorouracil, epirubicin, cyclophosphamide
HER2: human epidermal growth factor receptor 2
MDS: myelodysplastic syndrome
MRI: magnetic resonance imaging
Ph: Philadelphia
RT-PCR: real-time reverse transcriptase polymerase chain reaction
TN: triple negative
TNM: tumor nodes metastasis
VEGF: vascular endothelial growth factor
